# Effective application of indocyanine green for visualization of the thoracic duct during video-assisted thoracoscopic surgery for chylothorax: a case report

**DOI:** 10.1186/s44215-023-00098-3

**Published:** 2023-08-01

**Authors:** Teppei Hashimoto, Toshihiro Osaki, Soichi Oka, Hiroyuki Ueda

**Affiliations:** 1 Department of Thoracic Surgery, Kokura Memorial Hospital, 3-2-1, Asano, Kokurakita-Ku, Kitakyuusyu-Shi, Fukuoka 802-8555 Japan; 2 Department of Radiology, Kokura Memorial Hospital, 3-2-1, Asano, Kokurakita-Ku, Kitakyuusyu-Shi, Fukuoka 802-8555 Japan

**Keywords:** Idiopathic chylothorax, Indocyanine green, Near-infrared light, Thoracic duct ligation

## Abstract

**Background:**

Chylothorax is an accumulation of typically milky-appearing lymphatic fluid within the pleural cavity. High-output chylothorax may lead to a severe risk of death if not treated promptly. Several studies have reported the effectiveness of indocyanine green (ICG) for chylothorax following lung cancer surgery, esophagectomy, and congenital and recurrent idiopathic chylothorax. Here, we report a case of a successful treatment of idiopathic chylothorax in an adult by identifying and ligating the thoracic duct (TD) using fluorescent thoracoscopy with inguinal lymph node injection of ICG.

**Case presentation:**

A 79-year-old man with nephrotic syndrome presented with a massive right pleural effusion. Based on the pleural effusion examination, the patient was diagnosed with idiopathic chylothorax. He underwent 26 days of conservative treatments, including intercostal chest tube drainage, subcutaneous injection of a somatostatin analog (octreotide), and diet control (low-fat diet, fasting). However, the conservative treatments failed. Therefore, thoracoscopic TD ligation using a combination of ICG and near-infrared (NIR) light was performed. Ultrasound-guided inguinal lymph node injection of ICG was performed before thoracoscopy. Although standard-mode thoracoscopy could not identify leakage points, when observed under NIR light, the TD could be detected using fluorescence contrast. The TD was clipped to the deepest level on the thoracic side. Furthermore, a fluorescence hotspot of ICG on the cranial side of the clipped TD, likely a leakage point, was confirmed, and the lesion was clipped. ICG fluorescence did not disappear during surgery. The thoracic tube was removed on postoperative day 7. To date, chylothorax has not recurred.

**Conclusions:**

We report the effectiveness of intraoperative NIR fluorescence with ICG in identifying the TD’s running. This technique can lead us to identify and ligate the TD with assurance and accurately treat chylothorax.

## Background

Chylothorax results from accumulation of chyle in the pleural space caused by disruption or obstruction in the flow of lymph along the thoracic duct (TD) [1]. It is a potentially life-threatening complication [2, 3] and sometimes forces patients to undergo surgery intervention when conservative treatment is ineffective. A clear intraoperative recognition of the TD is the most reasonable therapeutic approach to treat chylothorax. However, intraoperative identification of the TD course or leakage site is often difficult.

Intraoperative indocyanine green (ICG) with near-infrared (NIR) fluorescence is an emerging technique for the visualization of the TD course and chylous leakage. Several studies have reported the safety and usefulness of NIR fluorescence imaging with ICG for treating chylothorax [4–6]. Here, we report a case of idiopathic chylothorax treated with identifying and ligating the TD using ICG-enhanced fluorescence during video-assisted thoracoscopic surgery.

## Case presentation

**Fig. 1:**
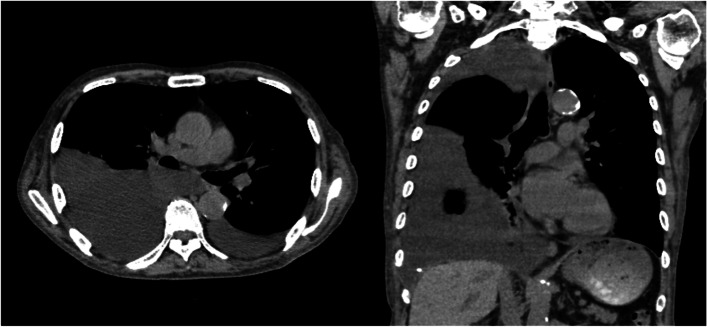
Computed tomography scan showing a uniform right massive pleural effusion

A 79-year-old male, recently diagnosed with nephrotic syndrome caused by focal segmental glomerulosclerosis refractory to steroids and immunotherapy, without any other comorbidities, presented with dyspnea on exertion. Chest radiography revealed right pleural effusion. Contrast-enhanced computed tomography (CT) showed gross right pleural effusion (Fig. 1). Furthermore, therapeutic thoracocentesis and pleural fluid analysis were performed. The pleural fluid was milky white in appearance, and pleural fluid analysis revealed a triglyceride concentration of 192 mg/dl, consistent with chylothorax. There was no history of recent surgery or catheter insertion; however, he fell at home and injured the right side of his back 2 days prior. CT showed no rib fractures, and the pain was mild. Additionally, he had no history of surgery, and the possibility of malignancy or lymphangioleiomyomatosis was not observed; thus, idiopathic chylothorax was diagnosed.

**Fig. 2:**
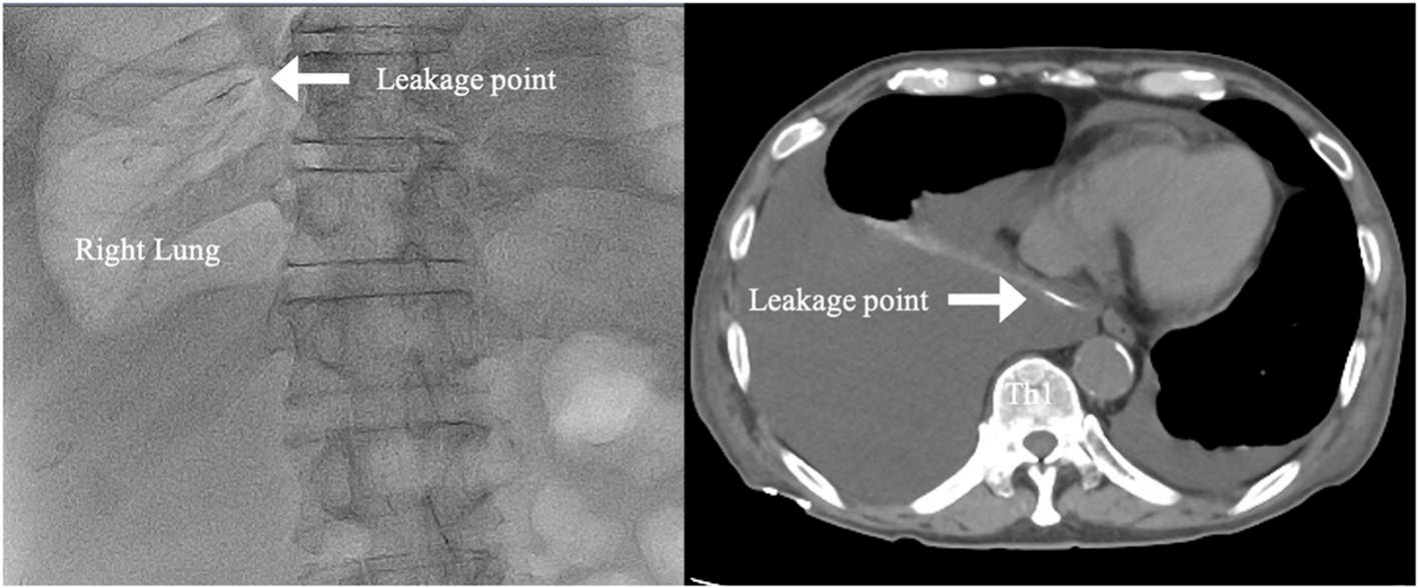
Intranodal lymphangiography showing leakage of Lipiodol from the thoracic duct into the right thoracic cavity. With the patient in the supine position, an ultrasound-guided right-sided inguinal lymph node injection of 11-ml Lipiodol was performed to follow the flow of contrast by fluoroscopy (Fig. 3). Approximately 20 min later, the injection of Lipiodol into the lymphatic vessel was confirmed under fluoroscopy. Immediately, CT was taken, which showed leakage of Lipiodol from the thoracic duct at the level of Th 11–12

First, the patient underwent conservative treatment, including drainage of the pleural cavity, diet control (complete oral intake cessation and a low-fat diet), and drug therapy (somatostatin analog, octreotide) for 26 days. In addition, lymphangiography revealed leakage of Lipiodol from the TD at the level of Th 11–12 (Fig. 2). Eventually, the chyle recurred after resuming a low-fat diet with massive pleural drainage, and the chest drain output remained as high as 2000 ml/day. Thus, we concluded that conservative treatment was ineffective and surgical intervention was required.

**Fig. 3:**
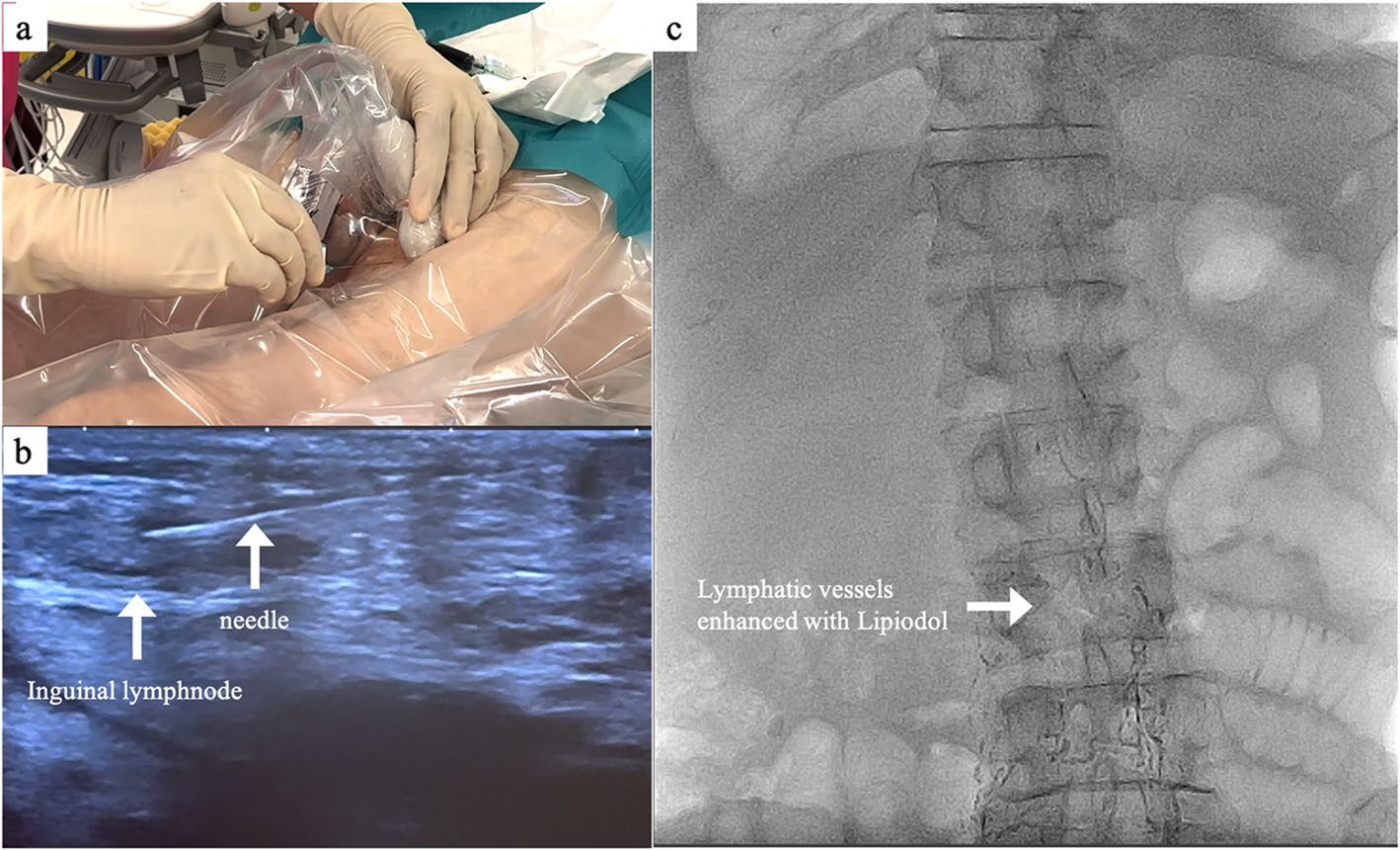
Ultrasound-guided inguinal lymph node administration of indocyanine green. After general anesthesia induction, with the patient in the supine position, an ultrasound-guided left-sided inguinal lymph node injection of Lipiodol was performed. Under fluoroscopy, Lipiodol flow injected into the lymphatic vessels was confirmed

After general anesthesia induction, with the patient in the supine position, an ultrasound-guided left-sided inguinal lymph node injection of 8-ml Lipiodol was performed to follow the flow of contrast by fluoroscopy (Fig. 3). Second, after confirming the injection of Lipiodol into the lymphatic vessel under fluoroscopy, 0.5 mg/kg of ICG (Diagnogreen; Dai‐Ichi Sankyo Pharma, Tokyo, Japan) diluted in 5 mL of physiological solution was injected using the same method. These steps required approximately 20 min. Subsequently, the patient was placed in the contralateral decubitus position and operated on under single-lung ventilation. Three 2-cm skin incisions were created in the sixth, ninth, and eighth intercostal spaces on the anterior, middle, and posterior axillary lines, respectively. A 30° camera with near-infrared (NIR) light acquisition and overlay technology was employed (The VISERA ELITE II System (Olympus Medical Systems, Tokyo, Japan) or 1688 Advanced Imaging Modalities (AIM) 4 K platform (Stryker Japan K.K., Tokyo, Japan)).

**Fig. 4:**
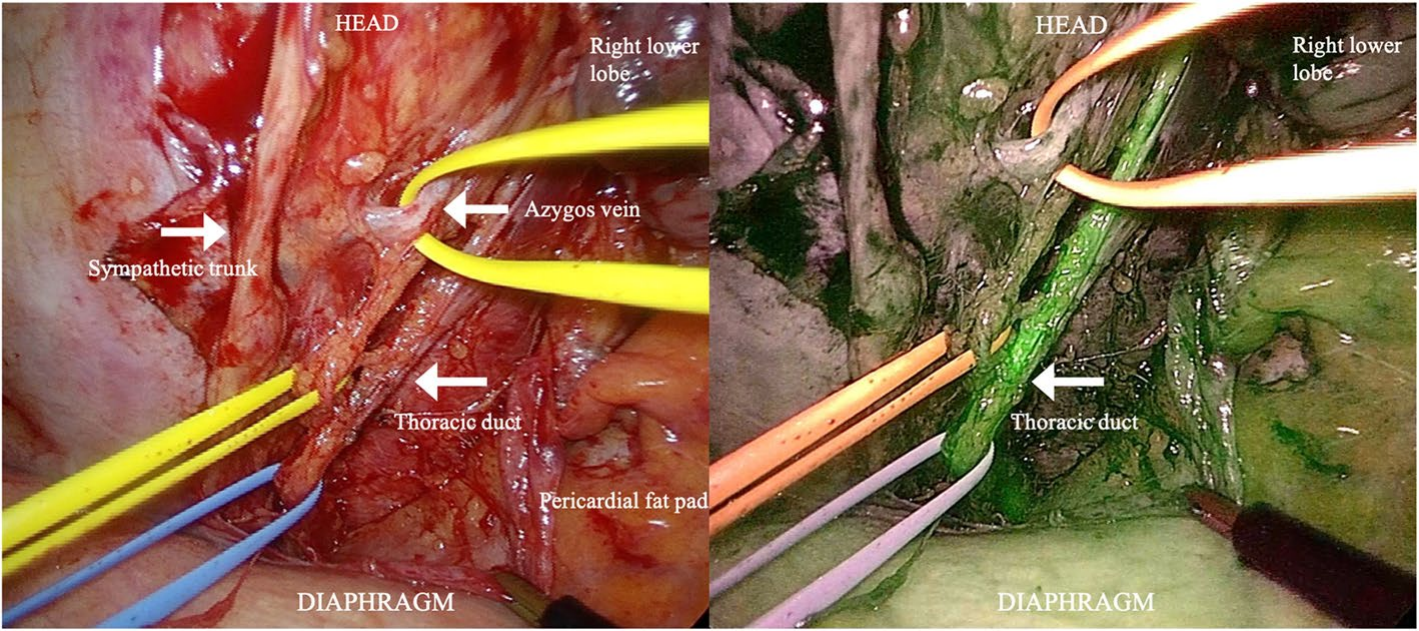
Near-infrared fluorescence contrast with indocyanine green (ICG) showing the thoracic duct. Near-infrared fluorescence imaging with ICG clearly visualized the thoracic ducts, which were distinct from the surrounding structures (sympathetic trunk, azygos vein, and their branches) and easily recognizable

**Fig. 5:**
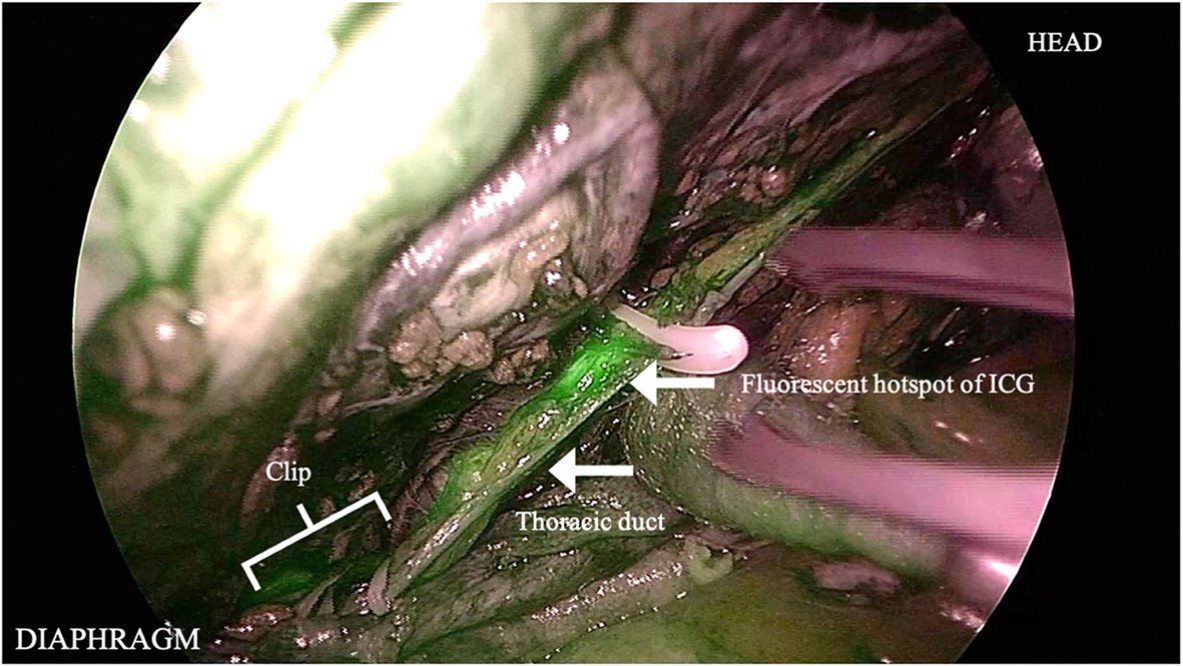
Near-infrared fluorescence hotspot enhanced with indocyanine green (ICG) on the thoracic duct. The fluorescence hotspot was detected at the clipped downstream thoracic duct (TD). If the hot point reflected ICG leakage from the fistula, we clipped the downstream side of the hot spot because the upstream side of the TD had already been clipped. During the operation, chylous leakage from the hot spot was not identified despite the administration of high-fat meals

Thoracoscopic observation detected no chylous leakage point. Although we administered high-fat ice cream intraoperatively through the gastric tube, we could not identify the leakage point. Subsequently, we thoracoscopically dissected the mediastinal pleura and taped the ligaments, similar to the TD and azygos veins. Approximately 100 min after the ICG was injected into the inguinal lymph node, when observed again under NIR light, the TD could be identified using fluorescence contrast; then, it was clipped at the deepest level of the thoracic side (Fig. 4). In addition, we confirmed the fluorescence hotspot of ICG on the cranial side of the clipped TD, possibly a leakage point, and clipped the TD at the cranial side (Fig. 5). The leakage point was consistent with the point identified during preoperative lymphangiography.

ICG fluorescence did not disappear during the thoracic surgery. Moreover, no significant blood loss or intra-operative complications were observed. Oral feeding was restarted with a low-lipid-content diet on postoperative day 1. On postoperative day 3, the pleural effusion volume was 1045 mL; however, on other days, the postoperative pleural effusion volume ranged from 5 to 230 ml. The chest tube was removed on postoperative day 7. The patient recovered with no further adverse events; however, owing to the initiation of hemodialysis, he was discharged on postoperative day 42.

## Discussion and conclusions

We reported the successful treatment of idiopathic chylothorax by identifying and ligating the TD using fluorescent thoracoscopy with ICG.

The key to successful TD ligation is upstream ligation of the TD leakage site. However, identification of the anatomical structure of the TD may be challenging because of the high variability of its course and presence of traumatized tissues around the spilling area, especially in postoperative chylothorax [7]. It has been reported that the typical pattern of the TD is observed in only about 50% of people [8]. In addition, it is known that there are lymphatic vessels in the thoracic cavity that flow directly into the TD [9].

To locate a chyle leak, lymphoscintigraphy with technetium-99 m [10] or magnetic resonance-thoracic ductography [11] can be considered; however, this method does not allow real-time imaging or pinpointing of the exact leakage site. Moreover, a preoperative oral ingested heavy cream or olive oil method may be used to identify thoracic lymph ducts [12–14]; however, this method provided poor contrast results in comparison with ICG fluorescence lymphography in an animal model [14].

NIR fluorescence imaging with inguinal injection of ICG has been reported to be effective for the treatment of chylothorax after lung cancer surgery [4], esophagectomy [5], congenital chylothorax [6], and recurrent idiopathic chylothorax [15] as a simple and safe method with very high detection sensitivity of the TD.

NIR fluorescence imaging with ICG provides real-time visual confirmation of running of the TD and lymphatic fluid leakage from the TD [16]. The second advantage is that we observed the fluorescence of the TD using ICG for a long time. Vecchiato et al. [17] have reported that the fluorescence effect of ICG did not disappear during surgery and remained effective for a long time. The median operative time was 258 min (range, 221–506 min). In the present case, ICG fluorescence was visible during surgery. The time from the inguinal lymph node injection of ICG to the completion of thoracic operation was 164 min.

The third benefit of intraoperative NIR fluorescence imaging with ICG is that it can be used in conjunction with conventional methods; administering heavy cream or olive oil [14].

In our case, chylous leakage was not confirmed with intraoperative high-fat ice cream oral administration; however, the TDs were clearly distinct from the surrounding tissue and easily recognizable using NIR fluorescence imaging with ICG. Furthermore, Chang et al. [6] have reported that a fluorescent hotspot coincided with a chylous leakage point, and after ligation, there was no further accumulation of lymphatic ICG. In this case, a fluorescence hotspot of ICG was observed downstream of the TD, which may have been a chylous leakage site. Therefore, we clipped the TD at the cranial side and at the deepest level of the thoracic side.

In conclusion, intraoperative NIR fluorescence imaging using an inguinal lymph node injection of ICG can provide an effective visualization of the TD course. This technique could help identify and ligate the TD with assurance and precisely treat chylothorax.

## Data Availability

Not applicable.

## Abbreviations

ICG: Indocyanine green
TD: Thoracic duct
NIR: Near-infrared
CT: Computed tomography
